# Work Ability among Upper-Secondary School Teachers: Examining the Role of Burnout, Sense of Coherence, and Work-Related and Lifestyle Factors

**DOI:** 10.3390/ijerph17249185

**Published:** 2020-12-09

**Authors:** Petr Hlaďo, Jaroslava Dosedlová, Klára Harvánková, Petr Novotný, Jaroslav Gottfried, Karel Rečka, Markéta Petrovová, Bohumil Pokorný, Ilona Štorová

**Affiliations:** 1 Faculty of Arts, Masaryk University, 602 00 Brno, Czech Republic; dosedlova@mail.muni.cz (J.D.); harvankova@phil.muni.cz (K.H.); novotny@phil.muni.cz (P.N.); marketa.petrovova@mou.cz (M.P.); pokorny@agemanagement.cz (B.P.); 2 Faculty of Social Sciences, Masaryk University, 602 00 Brno, Czech Republic; jaroslav.gottfried@mail.muni.cz (J.G.); reckak@mail.muni.cz (K.R.); 3 Employee Preventive Care Clinic, Masaryk Memorial Cancer Institute, 656 53 Brno, Czech Republic; 4 Age Management z.s., Orlí 27, 602 00 Brno, Czech Republic; storova@agemanagement.cz

**Keywords:** work ability, burnout, sense of coherence, workload, aging teachers

## Abstract

Maintaining and promoting teachers’ work ability is essential for increasing productivity and preventing early exit from the teaching profession. This study aimed to identify the predictors of work ability among upper-secondary school teachers and examine the mediating role of burnout. A large and diverse group of Czech upper-secondary school teachers was surveyed to address this goal. The sample comprised 531 upper-secondary school teachers (50.0 ± 9.94 years, 19.9 ± 10.62 in the teaching profession, 57.6% females). Relatively greater empirical support was found for the effects of burnout, sense of coherence, work–life balance, and perceived relationships in the school environment on work ability than for the impact of age, homeroom teacher duties, workload, and caring for elderly relatives. Furthermore, burnout served as an important mediator of the relationship between sense of coherence and work ability. Teachers with a higher sense of coherence are thus better able to cope with adverse work circumstances and identify and mobilize internal and external resources to prevent professional exhaustion and the subsequent decline in work ability. The study can guide interventions on the work ability of teachers.

## 1. Introduction

Teachers play a crucial role in students’ development and the educational process. However, teaching is an increasingly demanding profession that faces many intrapersonal, interpersonal, organizational, and administrative obstacles, hurdles, and challenges [1]. The reasons why some teachers at the productive age leave this profession include the high demands of teaching and the limited individual preconditions for their fulfillment, as well as aging, since older teachers consider—and often take—early retirement [2]. It is important to address the issues related to motivating teachers to stay in the profession, especially the societal context characterized by the aging population. Aging affects the teaching profession as well, and many education systems have already been facing, or will have to face the challenge of teacher shortages in the upcoming years [2]. At the international level, several strategies and approaches have been discussed to address the problem of the decreasing number of teachers. One possible strategy to encourage aging teachers to stay in the labor force involves improving or supporting the maintenance of their work ability [3].

### 1.1. Theories and Findings Regarding Work Ability with an Emphasis on Teachers

Work ability is defined as a balance between personal resources and work characteristics [4] or individual capacity to complete required work tasks and cope with the job demands successfully [5]. The work ability concept was proposed to identify whether individuals are able to continue to meet the physical and psychosocial requirements of their profession. As such, work ability answers the fundamental question: “can a person fulfill the basic and essential functions required for a given job?” [6] (p. 21). The theoretical basis of the concept of work ability lies in the stress–strain model, which emphasizes the importance of human interaction and determinants of the work environment [7]. The model is based on the assumption that work ability is the result of the ways in which individuals perceive job demands and evaluate their abilities related to dealing with them [8]. Specifically, work ability resides at the intersection between physical and mental health, functional capacities, qualification, professional competencies, attitudes and motivation on the one hand and working conditions, work content and demands, and environmental factors on the other hand [9,10]. Therefore, the work ability concept is based on the interconnection of objective health-related and subjective (perceived) aspects of work ability [11]. Although work ability focuses on the perception of abilities, similar to self-efficacy, these are two different concepts. Self-efficacy is distinctly motivational, with additional emphasis on taking action and allocating resources to accomplish goals. In contrast, work ability focuses on meeting the fundamental demands of the job [6].

In the recent literature (for more detail, see [6,11]), work ability has been theoretically anchored in the framework of the job demands–resources model (JD-R) [11,12,13,14,15], selection, optimization, and compensation theory (SOC) [16], and socioemotional selectivity theory (SST) [17]. First, the JD-R model [11,12,13,14,15] describes the relationships between work characteristics (job demands and job resources) and work outcomes (health, well-being, motivation, work performance). Job demands (e.g., workload) refer to physical, psychological, social, or organizational characteristics that require sustained physical or mental effort or skills and are associated with physical and psychological costs. In contrast, job resources (e.g., supervisor and co-worker support) are structural and relational aspects of the job that help achieve work goals, reduce job demands, and encourage personal growth and development [12]. According to this framework, two psychological processes (namely health impairment process and motivational process) explain work outcomes. Recent conceptualizations of the JD-R model have integrated personal resources (e.g., optimism, self-efficacy, self-esteem) that individuals can draw upon to meet their job demands. However, personal resources can fulfill different roles in the JD-R model [15]. Since work ability is conceptualized as a balance between personal resources and job demands [4], the revised version of the JD-R model represents a suitable theoretical framework to better operationalize the work ability construct. Work ability can be incorporated into the JD-R model as an outcome influenced positively by job and personal resources and negatively by job demands [6]. Second, from the lifespan developmental perspective, the SOC theory [16] explains how people cope with physical and mental aging. According to the SOC theory, the precondition for successful aging is selection, optimization, and compensation. Selection means that the hierarchy of goals is redefined due to physical and mental decline, and such goals are chosen that allow the individual to balance personal resources and demands. Optimization is the process of obtaining, developing, and coordinating the use of personal resources to meet the selected goals. Compensation refers to the application of alternative means or the utilization of aids to compensate for age-related losses. Developmental changes that occur during aging can cause a mismatch between ability and job demands, and adaptive strategies incorporated in the SOC theory can explain differences in work ability. Third, the SST [17] helps explain the shift in personal goals and behaviors over time as people age. More specifically, younger people perceive the future as more distant; therefore, they value future investments and focus more on goals linked to knowledge acquisition, career planning, and new social relationships. On the other hand, older people recognize that they have relatively little time remaining; hence, they frequently focus on current and emotionally meaningful relationships and goals. Thus, this theory may explain why certain interventions (e.g., supervisor or co-worker support) may be more effective in supporting work ability at one stage of life rather than another. (Note. Cadiz et al. [11] conceptually integrated work ability with organizational (JD-R) and lifespan development (SOC, SST) theories).

Although Cadiz et al. [11]—in their review and synthesis of the work ability literature—drew attention to several problems associated with the originally atheoretical nature of the construct, a large body of research has explored different aspects of work ability. The studies focused on work ability outcomes have indicated that low level of work ability may increase productivity loss at work [18], unemployment [19], short- and long-term sickness absence [20], and early retirement intentions, and predict premature retirement [19,21]. On the contrary, higher work ability among older workers has a positive influence on the desire to continue employment upon reaching retirement age [22]. Besides, work ability significantly predicts the quality of life in the physical, psychological, relational, and environmental domains [23]. Among teachers, work ability was found to be a significant and important predictor of current job satisfaction [24]. Since high job satisfaction decreased the desire to leave the job [25], work ability might contribute not only to job satisfaction but also to retaining teachers in the profession.

In recent years, several studies, meta-analyses, and reviews have been published to advance our knowledge of the antecedents of work ability [11,26,27]. However, distinguishing between antecedents and outcomes can be difficult. It has been found that work ability decreases rapidly with age [8,27,28,29] and physical health problems [30,31]. Czech research on gender has shown that women tend to have lower work ability [32]. However, the international findings on the relationship between gender and work ability are ambiguous [29,33]. Besides, lifestyle factors linked to risky health behaviors might decrease the levels of work ability. The lack of leisure-time physical activity, unhealthy eating habits, low sleep quality, and smoking may negatively affect work ability [34,35,36,37,38]. The findings on alcohol drinking habits are ambiguous. However, some research has shown a negative link between alcohol consumption and work ability [10]. Other research has either not shown this link [34] or shown that not drinking alcohol was associated with a declined work ability [39]. Contradictory findings have probably emerged due to the distinct operationalization of alcohol consumption in individual studies. Overall, high-risk alcohol consumption is likely to have a negative influence on work ability, while low and moderate alcohol consumption could even be beneficial [11]. The beneficial effect of low to moderate drinking on work ability can be clarified by a J-shaped relationship between alcohol consumption and health outcomes, as described previously [40]. Low to moderate alcohol consumption was in some studies associated with better health outcomes than non-drinking [41]. Thus, low to moderate drinking can be positively connected with objective work ability. However, alcohol consumption is also associated with several health risks; therefore, it cannot be considered a reasonable way to support work ability [42]. In the non-working area, caring for children or caring for elderly relatives [35,36] has a negative effect on work ability. Some findings on the relationship between lifestyle factors and work ability have also been demonstrated among teachers. It has been shown that the decline in the work ability of teachers is associated with limited engagement in physical activities in leisure time [11] as well as problems with sleeping and caring for children [31]. 

Prior research has outlined that some physical, mental, and psychosocial work-related demands and resources have either positive or negative effects on employee work ability [27,43]. Harmful aspects of the work environment, such as working in an unfavorable physical climate, working with poor work tools, and a restless work environment, are associated with a lower work ability [10,44]. Among teachers, it has been found out that their work ability is adversely affected by, for example, noise at work [45], and that the physical demands of work are a risk factor negatively associated with work ability, especially in the cohort of middle-aged teachers [46]. Furthermore, poorly organized work process, time pressure, fear of failure or mistakes at work [28], monotonous and uninteresting work, lack of freedom or autonomy [10,27] were found to be negatively related with work ability. Some work characteristics, such as high job demands or lack of discipline, have been shown to have a negative effect on the work ability of teachers as well [45].

Furthermore, the emotional demands of teaching have a significant effect on the work ability of teachers. Specifically, a previous study has found a relationship between pupils’ misbehavior and the reduced work ability of teachers [47]. Another study of teachers has shown that their work ability is negatively linked to the number of years in the teaching profession [31]. These findings suggest that more years of teaching may have an adverse effect on teachers’ work ability. On the contrary, the intrinsic aspect of the job, more specifically, the perceived meaning of work, is a positive predictor of teachers’ work ability [46]. 

Researchers have also identified the social environmental factors that may have positive and negative effects on work ability. Findings from previous studies indicated that supportive organizational climate, favorable interpersonal relations, instrumental and personal co-workers and supervisors support, and positive feedback from supervisors [48,49,50,51,52] are positively related to work ability, whereas experiences of workplace bullying [53] affect work ability negatively. Similarly, the beneficial effect of the above-mentioned social factors has also been reflected in teachers’ work ability. However, Sottimano et al. [46] revealed that especially young teachers benefit from favorable social ties. On the other hand, a Brazilian study [54] found that work ability is negatively associated with physical and verbal violence against teachers in the school. Moreover, previous research has highlighted the role of psychological resources (e.g., self-esteem, self-efficacy, self-confidence) in work ability [33,48,55].

### 1.2. Work-Related Stress and Burnout in the Teaching Profession 

Previous research has shown that teachers experience increased work pace and job demands [56]. Accordingly, teaching is considered a highly stressful profession, with stress due to high job demands and work in stressful conditions being associated with declining work capacity in teaching as well as in other occupations [49,57,58,59]. Work-related stress may contribute to the emergence and development of several physical and mental disorders [60,61,62,63], which have already been previously proven to be important determinants of low work ability [64,65]. 

The inability to cope effectively with job demands and chronic work stress may lead to burnout among teachers [47,65,66,67]. Burnout is usually defined as a psychological syndrome characterized by physical and emotional exhaustion, cynicism, and reduced professional efficacy [68]. Thus, in the work domain, burnout is manifested by cynicism, loss of purpose, professional idealism, fatigue, and a decline in work performance due to reduced physical vitality, emotional energy, and exhaustion [69]. Burnout among teachers has a negative effect on the school climate, teacher–student relationships, quality of teaching, and achievement of educational goals, and it may even result in the teacher’s decision to leave the profession [70,71]. 

The JD-R model assumes that burnout results from the presence of high levels of job demands and insufficient job resources [14]. The main causes of teachers’ burnout include: rigid administrative practices; pupil misbehavior; an unfavorable physical environment and social school climate; less direct support from the headteacher, supervisor, or co-workers; problems in cooperation with parents; and high demands of parents on teachers [14,47,70,72,73]. Burnout is associated with the poor performance of homeroom teachers [67] who are responsible not only for their teaching but also for class management and ensuring communication between the school and family. In research involving German teachers [74], burnout was associated with part-time employment. Teachers who worked on a part-time contract usually have a higher workload [74] and have to combine work requirements with taking care of family and household, which may lead to work–life imbalance and work–family conflict. The lifestyle factors of teachers, such as work–life balance, good sleep quality and duration, or non-hazardous alcohol consumption [75], are protective factors of burnout. Teachers who do not spend standard working hours at school may further suffer from limited social ties with their co-workers, limited integration into the teaching staff, and lower social support. Previous studies revealed that teachers who feel that they lack such support are more prone to burnout [76].

Furthermore, teacher–student, teacher–coworker, and teacher–supervisor relationships have a significant effect on burnout [77,78]. Demerouti et al. [12] provided strong and consistent evidence that high and unfavorable job demands (i.e., high work pressure, unfavorable physical environment, emotionally demanding interactions with clients) are primarily and positively related to the exhaustion component of burnout. In contrast, job resources (i.e., performance feedback, rewards, job control, participation in decision-making, job security, supervisor support) are primarily and negatively related to disengagement from work. However, a revised version of the JD-R model [79] provided a new perspective on burnout in the teaching profession by including personal resources components (e.g., personality characteristics such as extraversion and neuroticism, optimism, self-efficacy, self-esteem). Personal resources, defined in the JD-R model as resilience and environmental control, may reduce burnout directly or indirectly via contextual factors [79].

Although the association between teachers’ burnout and work ability has been established [64,80,81], the direction of the relationship between these variables is not clear. For example, while some studies showed that burnout was a significant predictor of low work ability [65,82], other studies found that work ability significantly affects burnout [83]. It can be concluded that previous empirical findings of significant and bidirectional relationships between work ability and burnout are in line with the conceptual integration model of work ability proposed by Cadiz et al. [11], where burnout can be treated either as an antecedent or as an outcome of work ability. In the present study, we considered burnout as a possible cause of lower work ability, according to the JD-R model. Based on the health impairment process [15], we assumed that high job demands and insufficient job resources might result in a higher strain (burnout). Burnout can then have an unfavorable effect on work ability, affecting two essential work ability components—health and well-being [79].

### 1.3. Sense of Coherence

The original JD-R model included only environmental work factors. However, human behavior is the result of an interaction between environmental factors and personality characteristics. Thus, the revised JD-R model was extended to include personal resources, defined as psychological characteristics or aspects of the self associated with resiliency [84]. Resilience is currently not understood as a personality trait but as a universal set of somatically, personality, and socially based resources that manifest in behavior and allow an individual, group, or society to prevent, minimize, or overcome the consequences of adverse (stressful) situations [85]. The variables that distinguish resistant from non-resistant individuals are referred to as buffer salutoprotective characteristics. These include self-regulation, self-efficacy, self-esteem, attribution style, mindfulness, locus of control, hardiness, sense of coherence, self-actualization, dispositional optimism, perceived social support, and others [85,86,87]. The JD-R model included many of these salutoprotective characteristics among personal resources [79]; however, the concept of a sense of coherence has not yet received sufficient attention.

The sense of coherence is defined as the extent to which an individual feels ubiquitous, stable, and, at the same time, dynamic confidence that (1) stimuli from both the external and internal environment are well-organized, predictable, and meaningful, (2) they have available resources to meet the demands placed on them by these incentives, and (3) these demands are perceived as challenges worth investment and effort [88]. This concept is understood either as a personality characteristic or as a coping style serving to adapt the individual to the changing external environment [89]. 

Research has shown that a higher sense of coherence among teachers is positively associated with the assessment of their competence, work–life balance, and perceived health [90]. A growing body of literature has confirmed the direct negative effect of the sense of coherence on burnout; therefore, individuals with a higher sense of coherence experience less occupational burnout [91,92,93]. It can be assumed that workers with a strong sense of coherence can better identify the nature of the stressor they face and select appropriate resources for the specific situation. In addition, a sense of coherence has been found to moderate the effect of job demands on burnout and serve as a protective function [92], while a moderate effect of a sense of coherence on the relation between work environment and burnout has not been found [94]. 

Little research has been conducted on associations between a sense of coherence and work ability. The study of unemployed individuals provided evidence for the relation between the sense of coherence and work ability [95]. In contrast, the sense of coherence did not significantly contribute to the work ability of women on sick leave [96]. From a theoretical perspective [11,13], in the present research, the sense of coherence is considered a personal resource that might positively affect burnout and work ability among upper-secondary school teachers.

### 1.4. Present Study

Although maintaining and improving the work ability of teachers is essential for increasing productivity and preventing early exit from the teaching profession, limited empirical research has focused on the work ability in this group of professionals. Therefore, the knowledge of which interventions may contribute to the work ability of upper-secondary school teachers is lacking. Considering these gaps, our study aimed to identify the predictors of work ability among teachers and examine the potential mediating role of burnout in the relationship between these predictors and work ability. We grounded our research in the JD-R model [12,13,15,79] to propose and examine predictors of teachers’ work ability. The JD-R model can be applied to a wide range of occupations, and it can be used to improve employees’ well-being and performance [13]. Furthermore, our research adopted the conceptual integration model of work ability from Cadiz et al. [11].

First, based on empirical findings presented in the literature review, we hypothesized that the predictors of low work ability among upper-secondary school teachers are older age, female gender, a higher number of years in the teaching profession, fixed-term contract, part-time contract, higher level of workload, increased homeroom teacher duties, unfavorable professional relationships, inappropriate healthy habits, unsteady work–life balance, and more caregiving responsibilities. Second, based on the JD-R model [12,13], we hypothesized that high job demands (operationalized as workload) and low job resources (operationalized as relationships in working life) predict burnout and, through the health impairment process, predict low work ability. Furthermore, the sense of coherence as personal resources can positively affect teachers’ burnout and work ability. Third, in the revised JD-R model [15,79], burnout is expected to mediate the relationship between job demands, job resources, and personal resources on the one side, and work ability on the other side. Thus, we hypothesized that burnout serves as a significant mediator of the associations between workload, relationships in working life, sense of coherence, and work ability. The present study is unique in that it examined both sense of coherence as a predictor of work ability and clarified the role of burnout in the relationships between sense of coherence, workload, relationships in working life, and work ability. In this study, a multidisciplinary approach incorporating psychology, andragogy, and occupational medicine was applied. 

Upper-secondary school teachers are suitable for the present study because compared to primary and lower-secondary school teachers, this group is considerably diversified (e.g., teachers of general subjects, vocational subjects, practicum, vocational training, artistic subjects) with varied job demands [97]. Likewise, the Czech Republic is well suited to address the proposed research aims because, in the last few decades, significant changes have occurred in the age structure of the population. Although aging affects all Czech teachers, upper-secondary school teachers are more affected compared to primary and lower-secondary school teachers. Recent statistics have shown that the average age of upper-secondary school teachers of general subjects, vocational subjects, and practical training was 47.3 years, 50.1 years, and 49.5 years, respectively. The highest proportion of teachers was in the age category 55–59 (19.9%), and the number of teachers in higher age categories was steadily increasing. The Organisation for Economic Co-operation and Development (OECD) report [2] pointed out that 39% of upper-secondary teachers are aged 50 or over. Furthermore, over the past decade, the number of upper-secondary school teachers decreased by 19.1%, while the interest in teaching among graduates of the teacher preparation program remains low [98,99].

## 2. Methods

### 2.1. Participants and Procedures

A cross-sectional correlational study was conducted among teachers at general and vocational upper-secondary schools in the South-Moravian Region of the Czech Republic. The headteachers of all upper-secondary schools (ISCED (International Standard Classification of Education) 344, 353, 354) in the region mentioned above were approached with a formal request to allow us to carry out research related to teachers at their educational institution. The consent was granted by 15 schools; the questionnaires were administered in group settings (usually about 10–15 teachers) in a school building by two trained administrators who provided the participants with the necessary assistance. The data collection took place from March to June 2019 using paper-and-pencil questionnaires. 

This study comprised 531 upper-secondary school teachers (57.6% females). The mean age of the participants was 50.03 years (*SD* = 9.94), while the average time spent in the teaching profession was 19.91 years (*SD* = 10.62). Moreover, 88.2% had a permanent contract, 83.6% worked in a full-time job, 49.1% were homeroom teachers, 37.9% cared for children at home, and 17.9% cared for elderly relatives. Additionally, 238 (44.8%) participants worked as teachers of general subjects, 199 (37.5%) as teachers of vocational subjects, 67 (12.6%) as teachers of vocational training, 33 (6.2%) as teachers of practicum, and 3 (0.6%) as teachers of artistic subjects (note that some teachers in the sample had multiple teacher roles).

### 2.2. Measures

#### 2.2.1. Work Ability

Work ability was assessed using the Czech version of the Work Ability Index (WAI) [32,100]. This self-reported instrument combines a subjective assessment of one’s ability to cope with the physical and psychosocial demands of work with information about diagnosed diseases, functional limitations, incapacity for work, and psychological resources. The WAI includes seven dimensions that assess the following areas: (*i*) current work ability compared with the lifetime best; (*ii*) work ability in relation to the demands of the job; (*iii*) number of current diseases (subjective appraisal); (*iv*) estimated work impairment due to diseases; (*v*) sick leave during the past 12 months; (*vi*) prognosis of work ability two years from now; (*vii*) mental resources. While the original WAI contains a list of 51 diseases to assess the third dimension, we used a shorter version comprising 14 disease groups. Based on these seven dimensions, we calculated the overall Work Ability Index (WAI) score that ranges from 7 to 49 points, with higher scores indicating higher work ability. Furthermore, the WAI questionnaire has been found to have acceptable test–retest reliability [101]. Despite extensive use of the WAI, there are concerns related to the construct validity and psychometric qualities of this instrument [11]. The factor structure of the WAI is particularly problematic. While most studies supported the one-dimensionality of the instrument [19], other studies reported a bi-dimensional factor structure for the WAI [102]. In our sample, a one-factor CFA model of the WAI fit the data poorly: χ^2^(27) = 198.84, *p* < 0.001, CFI = 0.847, RMSEA = 0.116 (90% CI (0.101; 0.131)), SRMR = 0.067. Therefore, we tested a hierarchical model inspired by the best fitting model in Freyer et al. [102] with one second-order factor (general work ability) and three interpretable first-order factors: subjective work ability (saturating items 1, 2, and 6), ill-health (items 3, 4, and 5), and mental resources (items 7a, 7b, and 7c). This model provided a good fit: χ^2^(24) = 47.01, *p* = 0.003, CFI = 0.979, RMSEA = 0.045 (90% CI (0.026; 0.064)), SRMR = 0.032. The McDonald’s omega total (Ω_t_) reliability of the WAI total score, which is suitable even for multidimensional measures, was 0.75 in the present study, indicating acceptable internal consistency. Despite the two-factor structure fitting our data better, we chose to work with the total scale score to enhance the reliability because of a low number of items per factor.

#### 2.2.2. Burnout 

Burnout was measured by the Czech version of the Shirom–Melamed Burnout Questionnaire (SMBQ) [103]. The SMBQ is a 14-item inventory consisting of three subscales that measure physical exhaustion, cognitive weariness, and emotional exhaustion. In our sample, a CFA model with the presupposed three-factor structure showed a good fit: χ^2^(74) = 286.52, *p* < 0.001, CFI = 0.959, RMSEA = 0.074 (90% CI (0.065; 0.083)), SRMR = 0.031. The SMBQ items were measured on a 7-point Likert-type scale with response options ranging from 1 (never or almost never) to 7 (always or almost always). The total score is averaged by dividing by the number of items. A high score indicates high burnout. In the present study, the estimated internal consistency reliability of McDonald’s Ω_t_ of the total score was 0.95, indicating excellent internal consistency.

#### 2.2.3. Sense of Coherence

The sense of coherence was assessed with a short version of the Sense of Coherence scale (SOC-13) [88,104,105], which captures the participant’s view of life, perception of stressful situations, and the ability to use generalized resistance resources to maintain and improve one’s health. This multidimensional questionnaire consists of 13 items that are divided into three subscales: comprehensibility, manageability, and meaningfulness. Each item was measured on a 7-point scale ranging from 1 to 7 with two anchoring claims. Higher mean scores denote a stronger sense of coherence. Overall, many studies have shown that the SOC-13 is a reliable, valid, and cross-culturally applicable instrument [106]. In this study, following the recommendations of the author of the questionnaire [104], we worked with a one-factor model. In our sample, a one-factor CFA model fit the data acceptably: χ^2^(64, *N* = 520) = 197.19, *p* < 0.001; CFI = 0.920, RMSEA = 0.063 (90% CI (0.053; 0.073)), SRMR = 0.047. The SOC-13 scale showed good internal consistency, where McDonald’s Ω_t_ for the overall scale was 0.83.

#### 2.2.4. Professional Relationships

Professional relationships were assessed using a set of four items asking about the quality of relationships with people in working life, more specifically with co-workers at a horizontal level and with students, students’ legal representatives, and the headteacher at a vertical level. Participants answered items on a 4-point Likert scale ranging from 1 (poor) to 4 (excellent). The participants could also choose the option: “I can’t assess/I don’t want to answer”. An average was calculated from all the items. The estimated internal consistency reliability of McDonald’s Ω_t_ was 0.67, indicating low internal consistency.

#### 2.2.5. Workload

Participants were asked to assess the workload in areas of administrative burden, teaching duties, and non-teaching duties on a 4-point Likert scale ranging from 1 (low) to 4 (very high). An average was calculated from all items. McDonald’s Ω_t_ was 0.62, indicating low internal consistency.

#### 2.2.6. Lifestyle

To evaluate lifestyle, the authors constructed a short assessment tool, the Teacher Lifestyle Scale (TLS), which tries to capture the specifics of teachers’ daily routine. First, the authors examined studies and book chapters that had investigated or theorized about the teacher lifestyle and then created 15 items that were found to be crucial. Participants answered items on a 4-point Likert scale ranging from 1 (very untrue of me) to 4 (very true of me). They could also choose the option: “I can’t assess/I do not want to answer”. Exploratory factor analysis (EFA) was performed to investigate the factor structure of the instrument. Based on scree plot inspection, we specified a two-factor solution. The first factor explained approximately 26% and the second one about 10% of the observed variance. The estimated correlation between both factors was 0.35. Loadings from oblique geomin rotations and communalities are presented in Table 1. Items for the TLS were selected based on their loadings and an absence of salient cross-loading. Items 1, 2, 3, 4, and 6 represented the *work–life balance* factor. Items 10, 11, 12, 13, and 14 represented the *maintaining healthy habits* factor. Additionally, confirmatory factor analysis (CFA) was conducted only on the selected items. All items were specified to load only on the respective factor, but we also freed residual covariance between a pair of items with similarly worded (items 1 and 2, both mentioning “having enough time”). The CFA model showed a good fit: χ^2^(42) = 115.35, *p* < 0.001, CFI = 0.957, RMSEA = 0.059 (90% CI (0.046; 0.072)), SRMR = 0.039. All items had loadings greater than 0.50. Subsequently, the scores on both subscales were calculated as the average of the items, with higher scores indicating more appropriate work–life balance and healthy habits. The McDonald’s Ω_t_ reliabilities were 0.73 for the work–life balance subscale and 0.88 for the maintaining healthy habits subscale.

#### 2.2.7. Other Covariates 

In the questionnaire, the participants were asked about their demographic attributes (age, gender), work-related characteristics (number of years in the teaching profession, employment status, employment type, homeroom teacher), and caregiving responsibilities (childcare, caring for elderly relatives). Variables age and number of years in the profession were metric, while the remaining variables were dichotomous.

### 2.3. Statistical Analyses

Descriptive statistics were calculated first to demonstrate demographic, work-related, and other characteristics of participants, followed by correlations among study variables. Subsequently, a block-wise regression analysis was used to determine the predictors of work ability among upper-secondary school teachers. The predictors were grouped into three blocks based on the literature review and conceptual consideration. In the first step, work ability was regressed onto demographic variables (age, gender), work-related characteristics (years in the teaching profession, employment status, employment type, homeroom teacher, and workload), relationships in working life (relationships with students, co-workers, headteacher, and students’ legal representatives) and caregiving responsibilities (childcare, caring for elderly relatives). In the second step, a sense of coherence and lifestyle variables (work–life balance and maintaining healthy habits) were entered. In the third step, burnout was entered as the last regressor. Finally, the structural equation model (SEM) was employed to establish a comprehensive assessment of the relationships between sense of coherence, burnout, and work ability. We used the R environment for our analysis [107], relying on the following packages: tidyverse (version 1.3.0) [108] for general data manipulation and cleaning, lavaan (v. 0.6-2) [109] and semPlot (v. 1.1.1) [110] for estimating and plotting SEM, respectively, and psych (v. 1.8.4) [111] for exploratory factor analysis and reliability estimation.

### 2.4. Ethical Consideration

The Research Ethics Committee of the Masaryk University (EKV-2018-045) reviewed and approved this study’s procedures, which was carried out according to the committee’s recommendations. This study complied with the ethical standards of the Declaration of Helsinki and the ethical standards of the American Psychological Association (APA). The informed consent form, which was presented at the beginning of the data collection and obtained from all participants, stated that participation in the study was voluntary, unpaid, anonymous, and confidential.

## 3. Results

### 3.1. Correlations

The correlation matrix of the observed indicators is displayed in Table 2. The correlations were generally in the expected directions. The correlation between work ability and burnout was strong and negative. Furthermore, work ability correlated positively with a sense of coherence, work–life balance, relationships in working life, and maintaining healthy habits and negatively with the workload. In addition, a negative correlation was observed between burnout and a sense of coherence and between burnout and relationships in working life, whereas a positive correlation was noted between workload and burnout. 

### 3.2. Resources Predicting Work Ability

Using block-wise regression analysis, we examined the predictors of work ability. As seen in Table 3, several work-related characteristics and caregiving responsibilities predicted work ability in the first step (Model 1). The regression analysis indicated that upper-secondary school teachers assessed their work ability as higher if they had better job resources and better professional relationships with students, co-workers, headteachers and students’ legal representatives (β = 0.27, *p* < 0.001), perceived a lower level of workload (β = −0.19, *p* < 0.001), were not homeroom teachers (β = −0.10, *p* = 0.036), and were not burdened by caring about their elderly relatives (β = −0.09, *p* = 0.052). All the variables in Model 1 explained 14% of the variance in work ability, with the *R^2^*-value being a good representation of the amount of variance explained. In the second step (Model 2), predictors of work ability included lifestyle, psychological, and demographic variables. Teachers who reported higher level of work ability maintained a steady work–life balance (β = 0.23, *p* < 0.001), obtained a strong sense of coherence (β = 0.21, *p* < 0.001), were younger (β = −0.15, *p* = 0.027), reported better professional relationships (β = 0.14, *p* = 0.002), and were not homeroom teachers (β = −0.09, *p* = 0.046). Model 2 explained a moderate amount of additional variance. Specifically, the predictor variables explained 28% of the total variance in work ability. The final model (Model 3) added burnout as a predictor, which increased the variance explained to 16% (44% in total). This improvement is substantial. Thus, based on the final model, the strongest predictors of favorable work ability are low level of burnout (β = −0.54, *p* < 0.001) and younger age (β = −0.15, *p* = 0.008), followed by not being a homeroom teacher (β = −0.12, *p* = 0.003), and suitable work–life balance (β = 0.11, *p* = 0.018). It seems that burnout correlated with some of the previous predictors (Table 2), reducing their effects in the final model.

### 3.3. The Mediation Role of Burnout

**Figure 1:**
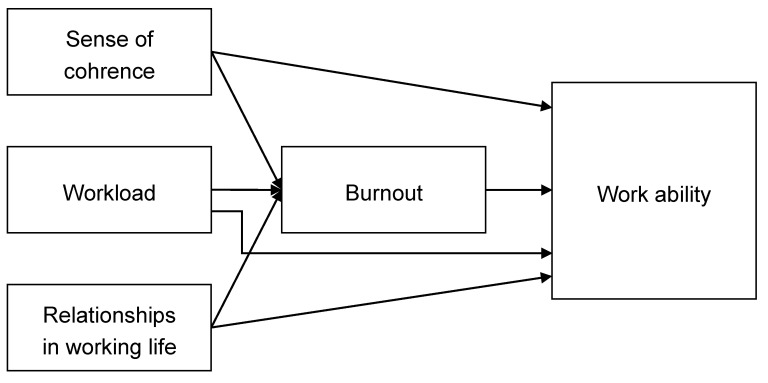
Proposed mediation model.

First, we estimated a baseline model in which all the latent variables representing work ability, burnout, sense of coherence, workload, and relationships in working life were allowed to correlate, that is, no regression paths were specified. The estimated model had an unacceptable fit to the data, χ^2^(67) = 272.19, *p* < 0.001, CFI = 0.895, RMSEA = 0.076 (90% CI (0.067; 0.086)), SRMR = 0.058. Accordingly, based on the analysis of modification indices and residual correlations, the model was revised (specifically, the item measuring mental resources dimension was excluded from the WAI because it is also saturated with the latent variable sense of coherence). The modified model had a good fit to the data, χ^2^(41) = 87.79, *p* < 0.001, CFI = 0.971, RMSEA = 0.046 (90% CI (0.033; 0.060)), SRMR = 0.034. Therefore, to test the mediation effect of burnout on work ability, we estimated a model in which sense of coherence, workload, and professional relationships predicted (i) burnout and (ii) work ability (Figure 1). The structural coefficients and indirect effects are reported in Table 4. The relationship between burnout and work ability (β = −0.98, *p* < 0.001) was very strong after controlling the effects of sense of coherence, workload, and professional relationships. The model explained 55% of the variance in burnout and 81% in work ability. We further examined the mediating role of burnout in work ability. The indirect effects of sense of coherence (β = 0.58, *p* < 0.001) and workload (β = −0.19, *p* = 0.002) on work ability through burnout were significant, whereas the indirect effect of relationships in working life (β = 0.15, *p* = 0.051) was not significant. The effect of workload was statistically significant, albeit small in magnitude. 

## 4. Discussion

This study aimed to extend the understanding of the relationships between upper-secondary school teachers’ work ability on the one hand and individual demographic characteristics, work-related attributes, professional relationships, lifestyle factors, and caregiving responsibilities on the other hand. Particular focus was on investigating the mediating role of burnout in work ability. 

This study’s findings are in line with previous research showing that older age is negatively related to teachers’ work ability. Although some studies have shown that functional aging does not necessarily have to be linked to chronological aging [112], a considerable number of studies have concluded that work ability decreases rapidly with increasing age [8,27]. In addition, individuals over the age of 50 experience a significant decline in work ability and show greater individual differences in this dimension [28,29]. Our results suggest that teachers’ work ability decreases as a function of age and that older teachers have reduced physical or intellectual capacities, which are two significant components of work ability [33]. The health impairment process proposed in the JD-R model could further clarify this finding [13,15]. Teachers are experiencing high job demands, the fulfillment of which is becoming increasingly difficult with aging. Specifically, job demands exhaust older teachers’ mental and physical resources, leading to decreased energy, health problems, and work ability. From the developmental perspective, aging is a continuous process of changing across the lifespan that requires individual adaptation. Therefore, our findings confirm that great attention should be paid to individually tailored interventions to promote and maintain teachers’ work ability throughout their careers. 

Consistent with the JD-R model of burnout [12] and other work ability studies [45,51,52], job demands and job resources, including workload, homeroom teacher duties, and professional relationships, were found to be significant predictors of work ability among teachers. The conceptual integration model of work ability proposed by Cadiz et al. [11] seems to clarify the effects of workload, homeroom teacher duties, and professional relationships on teachers’ work ability found in our research. Based on this model, job demands and resources (i.e., job demands, personal resources, structural job resources, and relational job resources) influence the lifespan development adaptation mechanism. As such, a long-term imbalance between demands and resources may negatively affect the work ability of teachers [113]. Our results uphold two of the JD-R model’s main principles: health impairment and motivational processes [13,15]. Unexpectedly, we did not find evidence to support the effects of time spent in the teaching profession, work status, and type of contract on work ability established in previous research [31,74]. First, we proposed that with the growing number of years in the teaching profession, work ability would decline due to energy depletion and health problems. Our results suggest that chronological time is not as crucial as age in the teacher profession; rather, the connection of time with the gradual acquisition of work experience is possibly even more vital. More specifically, experienced teachers are likely to be better able to cope with job demands compared to beginning teachers, for whom their entry into the profession is associated with an increased burden [114]. Another possible explanation is that only teachers who can handle demanding work requirements remain in the teaching profession, regardless of the length of their professional experience. However, this assumption should be further empirically verified. Second, we expected that the part-time contract would have a negative effect on work ability. Among employed persons, workers with a part-time contract had a lower work ability [115]. From previous research, we also know that part-time teachers usually have a higher workload, and they often feel to be somehow distant from their colleagues and receive diminished social support [74]. However, based on our findings, we can conclude that a part-time job does not seem to lead to exclusion from activities and social relationships that promote work ability. Moreover, employment status does not indicate how demanding and exhausting work is or whether teachers with lower work ability would choose part-time contracts. A part-time contract can even have a positive effect, as it provides an opportunity for active recreation and rest that are positively associated with work ability [27]. According to the SOC theory [16], part-time work could be a compensatory mechanism for older teachers, addressing the mismatch between personal resources and job demands. Third, we considered that a fixed-term contract would have a negative effect on work ability due to job insecurity. For example, previous research has shown that those who suffer from job insecurity experience negative work-related emotions, which might cause burnout, and report more health problems [116]. However, our study showed that employment type is not reflected in work ability among upper-secondary teachers.

Additionally, the subjective feeling of a heavy workload and the lack of time to meet work requirements may be appraised as threatening and result in strain [52]. In the JD-R model, high job demands are the most important antecedents of burnout symptoms [12]. Not surprisingly, we found that the heavy workload in administrative, teaching, and non-teaching duties is an important predictor of burnout. On the contrary, this study’s results did not support the assumption that unfavorable relationships in working life significantly affect teachers’ burnout, albeit the connection between interpersonal relationships at work and burnout has been well documented in the literature (e.g., [12,78]). However, our results might be affected by the tool used, which measured professional relationships as a whole, and did not distinguish between relationships with superiors, co-workers, students, and students’ legal representatives. The present study suggests that teachers’ relationships in the school environment may have different and even contradictory qualities and therefore need to be measured separately. 

Among non-work factors, caring for elderly relatives was a significant predictor of lower work ability, supporting our assumptions (e.g., [36]), while, surprisingly, caring for children was not [35]. Strong evidence suggests that long-term caregiving has a substantial effect on physical and mental health. For example, caregivers have more disease symptoms, physical limitations, and chronic conditions, and consistently higher rates of depression symptoms compared to non-caregivers [117]. Caring for elderly relatives thus fundamentally affects the health dimension of work ability. We also found that mainly aging teachers provide care for elderly relatives. Correspondingly, we believe that the physical and emotional demands associated with caring responsibilities, especially for aging teachers, may thus further intensify the decline in their work ability. However, not just aging teachers will have to face the challenges of demographic shifts in the future. As mentioned above, society is constantly aging; therefore, the number of people in need of care will increase, and caring will become one of the major non-work responsibilities for a significant proportion of teachers. Our research suggests that school leaders should seriously address the needs of teachers caring for elderly relatives and establish an effective workplace policy to help them balance their caring and teaching roles [118]. Our findings extend previous research on the positive role of lifestyle factors, showing that work–life balance positively affects work ability among teachers. It turns out that teachers’ work ability depends, to a large extent, on how they can balance teaching with other roles that make up their life systems. In the context of previous research, the promotion of teachers’ work–life balance seems to be important not only for their work ability but also for job and life satisfaction and mental health, which are closely related to work ability [119]. 

We further hypothesized that the long-term burden of the teaching profession might be associated with burnout [66], resulting in a decline in teachers’ work ability [65]. The present study provides substantial evidence that burnout in the teaching profession is linked to negative occupational consequences [47]. Our analyses showed that burnout was a statistically significant predictor of work ability with a strong negative effect. The given results are consistent with the approaches of Maslach and Leiter [120] in the United States and Schaufeli and Bakker [14] in Europe, who contrasted work ability and work commitment with burnout to describe situations in which employees engage in personal activities, which increase their feelings of efficiency. Burnout has an unproductive relationship with work, while work commitment is related to positive attitudes with life and work (i.e., meaningfulness, endurance, and responsibility). From this perspective, the results that the sense of coherence is a strong predictor of burnout and work ability are not surprising. Specifically, individuals with a higher sense of coherence had a reduced risk of burnout and a higher level of work ability. These results are consistent with previous literature [92,93,95]. This follows from the philosophical, psychological, theoretical grounding of burnout, the source of which is existential psychology, especially Frankl’s [121] logotherapy. When an individual doubts the meaning of one’s work or even the meaning of one’s existence, as is often the case in the more advanced stages of burnout, one enters a state of existential frustration accompanied by negative affectivity and, potentially, symptoms of depression [121]. Antonovsky’s [88] research has already shown that people with a greater sense of coherence cope with stressful situations more efficiently, perceive obstacles as challenges, and do not easily give up while under stress. The concept correlates not only with the subjective component of personal well-being but also with the perceived health and lower incidence of psychosomatic disorders [122]. A high degree of sense of coherence is also associated with lower emotional exhaustion [123], which is a significant relationship, considering that emotional exclusion is one of the basic manifestations of burnout. The current study also confirmed the mediating role of burnout in the relationship between the sense of coherence and work ability. A possible explanation may be that sense of coherence is generally activated in various types of difficult situations connected with the teaching profession to serve as a coping mechanism [124]. Therefore, teachers with a higher sense of coherence can cope with adverse work circumstances better as well as identify and mobilize internal and external resources to prevent professional exhaustion and the subsequent decline in work ability [125]. In addition to the mediating model discussed above, we further examined the mediating role of burnout in the relationship between workload and work ability, which was significant, but the observed effect was rather small. 

The present study incorporated a new combination of organizational and personal factors to explain burnout and work ability among upper-secondary school teachers. In addition, the findings provide new empirical evidence to support some parts of the revised JD-R model [14]. We established that job demands (workload) and personal resources (sense of coherence) affect strain (burnout) directly, and they affect work outcomes (work ability) directly and indirectly through burnout. These conclusions support the part of the JD-R model, which proposes that burnout mediates the relationship between job demands and health problems [14]. We also provide empirical evidence that burnout mediates the relationship between personal resources (sense of coherence) and work outcomes (work ability) among teachers. The finding that certain personal resources (sense of coherence) could be antecedents of strain (burnout) and work outcomes (work ability) contributes to the JD-R model. In the case of job resources (relationships in working life), empirical support for the JD-R model was found only via their direct influence on outcomes (work ability). Other relationships (direct effect of job resources on burnout and indirect effect of job resources on work ability through burnout) were unsupported.

## 5. Limitations, Future Directions, and Practical Implications

Our results and conclusions need to be considered in the light of limitations that can inform directions for future research. First, this study used a cross-sectional research design with self-reports. Thus, causal inferences and long-term effects of all covariates on work ability should be examined in the prospective longitudinally study to evaluate the effects over time and to confirm causal relationships. Second, we must bear in mind that our findings are based on self-reported measures and reflect how teachers perceive and evaluate themselves, their health, work, and social environment. The responses might also be upward biased due to social desirability effects.

Further, while processing data, we found that some participants were under-motivated to complete the questionnaires. We assume that the missing answers to the WAI questionnaire may have been due to the fear of disclosing confidential information. Previous research has already highlighted these obstacles, namely that the WAI asks about personal health information, which participants may not want to divulge to researchers [11]. Thus, teachers who conscientiously completed the questionnaires and whose responses were analyzed in our study could have distinct attributes related to work ability. Accordingly, work ability research should consider collecting the data in a safe, non-group setting or designing and validating a new comprehensive instrument for measuring work ability rooted in one of the established theories. Third, the present study focused on upper-secondary school teachers only, reducing the generalizability of our findings to other teaching and helping professions. Fourth, albeit data in the present study supported the hypothesis proposing the mediating role of burnout in the association between sense of coherence, workload, and work ability, the magnitude of the direct burnout effect on work ability was extremely high. We believe that this may be due to a partial interrelation between the constructs of burnout and work ability. Future research should conceptualize and differentiate the constructs of work ability and burnout explicitly with the aim to design and validate a new comprehensive work ability instrument based on the established theories. Finally, this study did not investigate other factors influencing work ability, which have been well established in the literature (e.g., alcohol consumption).

In addition to enriching the existing theories, our findings might have some practical implications for the maintenance and promotion of teachers’ work ability. Our study also showed that the predictors of work ability are reasonable workload, good professional relationships, and work–life balance. Following the JD-R model [12,14], we proved that resources that are functional in achieving work goals, coping with job demands, and supporting growth and development in both work and personal life influence work ability. All these aspects may be supported at the individual level within the personal and professional development courses for teachers but also at an organizational level in schools. Based on our findings, we recommend that individual intervention training programs for teachers focus on the cognitive, emotional, and behavioral aspects of stress management to promote a healthy lifestyle and health, time management, and work–life balance (including ways to harmonize job demands with caring for minor children or aging parents). Although we found that work ability decreases with age, targeted intervention programs should not be aimed only at aging teachers.

On the contrary, our research suggests that the work ability of all teachers should be supported regardless of their age, although the characteristics of different age groups of teachers need to be considered. As mentioned above, schools may also play an important preventive role in burnout and work ability. School management should implement actions aimed at monitoring and adjusting job demands and job resources to protect the work ability of teachers and allow a greater number of them to continue teaching in optimal conditions in the latter stages of their careers [126]. Our findings and the literature highlight the importance of modifying the job demands placed on teachers, supporting work–life balance in the workplace, creating a healthy and socially sensitive school environment focused on interpersonal relations and mutual social support, increasing teacher participation in decision-making, and setting a clear career path system. The concept of age management offers a systematic solution to support the work ability of all age categories of teachers [127]. It is also concerned with personnel management, focusing on the age, abilities, and capacity of each individual and the changes that each person undergoes during working life. This approach has the potential to help teachers manage work by considering their chronological and functional aging, as it takes into account changing health, competencies, needs, but also values, attitudes, and motivations. The aging of the workforce and the support of teachers’ work ability are expected to be of increased interest to not only school management but also national education systems. Monitoring work ability at the school level can thus provide important information about the workforce, not only for individual interventions but also for conceptual changes in the education system [6].

## 6. Conclusions

The present study highlighted the important role of burnout, which emerged as the strongest predictor of work ability among teachers. Furthermore, relatively greater empirical support was found for the effects of the sense of coherence, work–life balance, and perceived professional relationships on work ability than for the effects of age, homeroom teacher duties, workload, and caring for elderly relatives. Nevertheless, the significant effects of individual predictors were relatively small, except for burnout. Unlike other research, our study did not show the effect of adherence to healthy habits (e.g., physical activity, mental hygiene, healthy eating habits, cold water baths) on teachers’ work ability. 

## Figures and Tables

**Table 1:** Exploratory factor analysis (EFA) of the Teacher Lifestyle Scale—factor loading matrix.

Item	F1	F2	*h* ^2^
1. I have enough time for leisure activities	**0.76**	0.03	0.59
2. I have enough time for my family life	**0.73**	–0.03	0.52
3. I sufficiently relax and rest	**0.71**	0.09	0.55
4. I can arrange my daily program so that I do not get into time stress	**0.69**	0.01	0.48
5. I face long-term stress in my daily life	–0.64	0.45	0.42
6. I adapt the daily routine to my current health condition	**0.56**	0.14	0.38
7. At work, I often get into stressful situations	–0.51	0.30	0.25
8. My sleep is usually long enough and relaxing	0.46	0.17	0.29
9. I eat meals regularly	0.32	0.18	0.17
10. I practice mental hygiene	–0.01	**0.59**	0.35
11. I try to eat healthily	0.20	**0.45**	0.31
12. I regularly harden	0.19	**0.41**	0.25
13. I regularly do sports or other physical activity	0.26	**0.40**	0.30
14. I follow the recommended drinking regime	0.26	**0.35**	0.25
Sum of the squared factor loadings	3.66	1.45	
Total variance explained	0.26	0.10	

Note. χ^2^(64) = 339.98, *p* < 0.001, CFI = 0.846, RMSEA = 0.100 (90% CI (0.090; 0.109)), SRMR = 0.060. Bold font indicates the items used to calculate the total score of the corresponding factor.

**Table 2:** Correlation Matrix.

	1	2	3	4	5	6	7	8	9	10	11	12	13	14
1. Work ability	–													
2. Age	−0.063	–												
3. Gender	−0.037	−0.100 *	–											
4. Years in the teaching profession	−0.024	0.720 ***	0.054	–										
5. Employment status	0.005	−0.108 *	0.010	−0.132 **	–									
6. Employment type	−0.027	0.002	0.069	0.015	0.206 ***	–								
7. Homeroom teacher duties	−0.077	0.022	−0.133 **	−0.022	0.077	0.247 ***	–							
8. Workload	−0.191 ***	0.014	0.086 *	0.077	−0.074	−0.135 **	−0.176 ***	–						
9. Professional relationships	0.300 ***	−0.010	0.064	0.046	0.080	0.074	−0.021	−0.098 *	–					
10. Childcare	0.009	−0.291 ***	0.133 **	−0.218 ***	0.041	−0.039	−0.041	0.044	0.001	–				
11. Caring for elderly relatives	−0.070	0.149 ***	0.073	0.163 ***	0.013	0.007	−0.008	−0.003	0.005	−0.202 ***	–			
12. Sense of coherence	0.390 ***	0.034	−0.030	−0.011	0.006	0.022	0.006	−0.207 ***	0.344 ***	0.004	0.003	–		
13. Work–life balance	0.390 ***	0.213 ***	−0.046	−0.114 *	−0.035	−0.039	−0.005	−0.277 ***	0.219 ***	−0.121 **	−0.033	0.427 ***	–	
14. Maintaining healthy habits	0.274 ***	0.078	0.044	0.055	−0.023	−0.015	−0.014	−0.110**	0.228 ***	−0.066	0.0282	0.231 ***	0.460 ***	–
15. Burnout	−0.636 ***	−0.054	0.079	0.010	−0.004	−0.019	−0.062	0.290 ***	−0.349 ***	0.044	0.042	−0.586 ***	0.045	−0.031

Coding: Gender (0 = male, 1 = female), employment status (0 = full-time, 1 = part-time), employment type (0 = fixed-term, 1 = permanent), homeroom teacher (0 = no, 1 = yes), workload (scale 1–4; the higher values mean heavier workload), professional relationships (scale 1–4; the higher values mean better relationships), childcare (0 = no, 1 = yes), caring for elderly relatives (0 = no, 1 = yes), sense of coherence (scale 1–7; the higher values denotes the stronger sense of coherence), work–life balance (scale 1–4; the higher values mean more steady work–life balance), maintaining healthy habits (scale 1–4; the higher values mean more appropriate healthy habits). * *p* < 0.05, ** *p* < 0.01; *** *p* < 0.001.

**Table 3:** Linear regression for work ability (WAI) as dependent.

Predictors	Model 1			Model 2			Model 3		
	*b*	*SE_b_*	β	*b*	*SE_b_*	β	*b*	*SE_b_*	β
(Constant)	37.835 ***	3.295		27.884 ***	3.247		47.403 ***	3.363	
Age	−0.037	0.037	−0.071	−0.076 *	0.034	−0.145	−0.081 **	0.030	−0.154
Gender	−0.701	0.505	−0.066	−0.681	0.466	−0.065	−0.422	0.411	−0.040
Years in the teaching profession	0.013	0.034	0.026	0.026	0.032	0.054	0.040	0.028	0.083
Type of Contract	−0166	0.763	−0.010	0.031	0.705	0.002	0.170	0.621	0.010
Work status	−0.625	0.688	−0.044	−0.360	0.637	−0.025	−0.282	0.561	−0.020
Homeroom teacher duties	−1.049 *	0.500	−0.100	−0.922 *	0.461	−0.088	−1.236 **	0.407	−0.118
Workload	−1.655 ***	0.416	−0.187	−0.721 ^†^	0.401	−0.082	−0.144	0.358	−0.016
Professional relationships	3.587 ***	0.609	0.272	1.842 **	0.602	0.140	0.645	0.542	0.049
Childcare	−0.182	0.516	−0.017	0.113	0.478	0.011	0.214	0.422	0.020
Caring for elderly relatives	−1.230 *	0.630	−0.092	−1.013 ^†^	0.589	−0.076	−0.668	0.520	−0.050
Sense of coherence				0.944 ***	0.210	0.214	0.057	0.201	0.013
Work–life balance				1.908 ***	0.438	0.229	0.938 *	0.395	0.113
Maintaining healthy habits				0.762 ^†^	0.404	0.089	0.586	0.357	0.068
Burnout							−3.102 ***	0.281	−0.535
*R* ^2^	0.141			0.275			0.439		
adj. *R*^2^	0.120			0.252			0.420		
Δ*R*^2^				0.134			0.164		
*F*(df) for *R*^2^	6.91 (10, 422) ***			12.22 (13, 419) ***			23.32 (14, 418) ***		
*F*(df) for Δ*R*^2^				25.84 (3, 419) ***			121.85 (1, 418) ***		

Coding: Gender (0 = male, 1 = female), employment status (0 = full-time, 1 = part-time), employment type (0 = fixed-term, 1 = permanent), homeroom teacher (0 = no, 1 = yes), workload (scale 1–4; the higher values mean heavier workload), professional relationships (scale 1–4; the higher values mean better relationships), childcare (0 = no, 1 = yes), caring for elderly relatives (0 = no, 1 = yes), sense of coherence (scale 1–7; the higher values denotes the stronger sense of coherence), work–life balance (scale 1–4; the higher values mean more steady work–life balance), maintaining healthy habits (scale 1–4; the higher values mean more appropriate healthy habits). * *p* < 0.05, ** *p* < 0.01, *** *p* < 0.001, ^†^ *p* < 0.10. WAI: Work Ability Index.

**Table 4:** Structural coefficients and tests of indirect and overall effects on work ability.

Regression Part of the Model	*b*	*SE*_*b*_	*Z*	β
Regression with burnout as a dependent variable				
Sense of coherence	−0.491 ***	0.073	−6.767	−0.590
Workload	0.273 ***	0.084	3.237	0.192
Relationships in working life	−0.748 ^†^	0.399	−1.876	−0.153
Regression with work ability as a dependent variable (direct effects)				
Sense of coherence	−0.629 **	0.216	−2.907	−0.299
Workload	0.599 **	0.210	2.854	0.167
Relationships in working life	3.188 **	1.237	2.577	0.258
Burnout	−2.478 ***	0.255	−9.710	−0.981
Tests of indirect effects				
Sense of coherence	1.216 ***	0.246	4.948	0.579
Workload	−0.676 **	0.219	−3.081	−0.189
Relationships in working life	1.854	0.951	1.950	0.150
Tests of total effects				
Sense of coherence	0.587 **	0.192	3.063	0.279
Workload	−0.077	0.239	−0.321	−0.021
Relationships in working life	5.043 ***	1.461	3.452	0.409

** *p* < 0.01, *** *p* < 0.001, ^†^ *p* < 0.10. Not*e.* χ^2^(41) = 87.79, *p* < 0.001, CFI = 0.971, RMSEA = 0.046 (90% CI (0.033; 0.060)), SRMR = 0.034.
